# Acquired Alterations of Hypothalamic Gene Expression of Insulin and Leptin Receptors and Glucose Transporters in Prenatally High-Glucose Exposed Three-Week Old Chickens Do Not Coincide with Aberrant Promoter DNA Methylation

**DOI:** 10.1371/journal.pone.0119213

**Published:** 2015-03-26

**Authors:** Rebecca C. Rancourt, Karen Schellong, Raffael Ott, Semen Bogatyrev, Barbara Tzschentke, Andreas Plagemann

**Affiliations:** 1 Clinic of Obstetrics, Division of ‘Experimental Obstetrics’, Charité—University Medicine Berlin, Campus Virchow-Klinikum, Berlin, Germany; 2 Humboldt-University of Berlin, Institute of Biology, Berlin, Germany; University of Santiago de Compostela School of Medicine—CIMUS, SPAIN

## Abstract

**Background:**

Prenatal exposures may have a distinct impact for long-term health, one example being exposure to maternal ‘diabesity’ during pregnancy increasing offspring ‘diabesity’ risk. Malprogramming of the central nervous regulation of body weight, food intake and metabolism has been identified as a critical mechanism. While concrete disrupting factors still remain unclear, growing focus on acquired epigenomic alterations have been proposed. Due to the independent development from the mother, the chicken embryo provides a valuable model to distinctively establish causal factors and mechanisms.

**Aim:**

The aim of this study was to determine the effects of prenatal hyperglycemia on postnatal hypothalamic gene expression and promoter DNA methylation in the chicken.

**Methods and Findings:**

To temporarily induce high-glucose exposure in chicken embryos, 0.5 ml glucose solution (30 mmol/l) were administered daily *via* catheter into a vessel of the chorioallantoic egg membrane from days 14 to 17 of incubation. At three weeks of postnatal age, body weight, total body fat, blood glucose, mRNA expression (*INSR*, *LEPR*, *GLUT1*, *GLUT3*) as well as corresponding promoter DNA methylation were determined in mediobasal hypothalamic brain slices (Nucleus infundibuli hypothalami). Although no significant changes in morphometric and metabolic parameters were detected, strongly decreased mRNA expression occurred in all candidate genes. Surprisingly, however, no relevant alterations were observed in respective promoter methylation.

**Conclusion:**

Prenatal hyperglycemia induces strong changes in later hypothalamic expression of *INSR*, *LEPR*, *GLUT1*, and *GLUT3* mRNA. While the chicken provides an interesting approach for developmental malprogramming, the classical expression regulation via promoter methylation was not observed here. This may be due to alternative/interacting brain mechanisms or the thus far under-explored bird epigenome.

## Introduction

Overweight and obesity are continuously increasing in epidemic-like proportions as are the downstream health risks associated with developing diabetes and alterations typical for the Metabolic Syndrome [1, 2]. It is more and more accepted that early life maternal and other environmental experiences have distinct impact for the long-term offspring health through ‘programming/malprogramming’ of body functions during ‘critical periods’ of perinatal life [3, 4]. During neuronal development, hyperglycemia (*e*.*g*. through gestational diabetes) may cause long-lasting malprogramming of the central nervous regulation of body weight, food intake and metabolism, resulting in an increased ‘diabesity’ risk [5, 6]. Still unclear are the basic mechanisms behind perinatal 'malprogramming', made more challenging with the lack of animal models available to decipher singular risk factors irrespective of potential confounders and variables, as unavoidable in the complex placental mammalian mother-fetus-environment-interaction.

Because of its nearly ‘unaffected’ embryonic development, independently from the mother, the chicken provides an excellent model for investigations of pre- and perinatal developmental processes [7–9]. It allows highly standardized and controlled manipulations of pre- and perinatal environmental factors during distinct time windows of embryonic development. Physiological developmental pattern during late prenatal period of the chicken have similarity to that in mammals and human fetuses [9]. Recently there have been a growing number of respective studies involving chicken [10–16], especially with the availability and improved annotation of the chicken sequence/genome [17]. Furthermore, the chicken shows similar developmental modes and causes of obesity and related metabolic disorders [18–20]. Beyond genetic predispositions, environmental factors may induce disruption in the glucose-insulin-balance and increased adiposity in the chicken, too [21, 22].

Interestingly, the neuroendocrine regulation of energy balance in birds and mammals is also very similar [23, 24]. While in mammals, the mediobasal Nucleus arcuatus hypothalami (ARC) integrates hormonal and metabolic signals from the periphery, especially insulin, leptin, and glucose, to regulate metabolism, food intake and body weight [25, 26], its structural and functional equivalent in birds is the Nucleus infundibuli hypothalami (NI) [27, 28]. Insulin receptor (*INSR*), leptin receptor (*LEPR*) as well as glucose transporters (*GLUT*) have been identified in bird NI accordingly [10, 14, 29–33]. In mammals, persisting molecular as well as functional hypothalamic resistance to insulin and leptin were observed in consequence of perinatal overfeeding and, especially, materno-fetal hyperglycemia, obviously predisposing to lasting increased ‘diabesity’ risk [34–38]. Interestingly, more recent observations indicate that perinatally acquired alterations of respective promoter DNA methylation may contribute to this developmental malprogramming [39–42]. However, concrete causal and ‘disruptive’ factors remain unknown.

Therefore, this study was carried out to determine effects of prenatal high-glucose exposure on the postnatal hypothalamic expression of *INSR*, *LEPR*, and glucose transporters (*GLUT1* and *GLUT3*) in prenatally glucose-treated chickens as compared to NaCl-injected controls, and to evaluate related promoter DNA methylation pattern analyzed here for the first time in chicken.

## Materials and Methods

### Ethics Statement

All animal procedures were performed in accordance with the European Communities Council Directive (86/609/EEC) and were approved by the local animal welfare committee (G 0275/09; Lageso Berlin, Germany).

### Animal model and study design

Experiments were carried out in three-week-old juvenile chickens (*Gallus gallus f*. *domestica*), hatched from eggs incubated prenatally under different metabolic conditions. Eggs were obtained for research approaches from a commercial breeder (Lohmann Tierzucht GmbH, Cuxhaven, Germany). In the course of the 21 days of embryonic (‘fetal’) development, the eggs were incubated under standard conditions (temperature of 37.5°C, relative air humidity 70% to 90% during hatching period, and automatic turning). During days 14 to 17 of incubation, a ‘critical period’ in the development of the neuro-endocrine system of chicken embryos [9], the eggs were divided into two groups; injected daily with either 0.5 ml 0.9% NaCl solution (NaCl-treated control group, NaCl) or with 0.5 ml of a highly-concentrated glucose solution (30 mmol/l D(+) Glucose, Carl-Roth GmbH, Germany) to induce hyperglycemia in chicken embryos (glucose-treated group, Glc). For induction of hyperglycemia this high concentration was used because normal avian blood glucose level is approximately double that of healthy mammals [43]. Injections were applied *via* a catheter (BD Valu Set, needle Ø 27G) fixed on the eggshell and inserted into a vessel of the chorioallantoic membrane through a small square hole (3 mm each side), drilled to reach the blood vessel under the egg shell. After catheterization the hole was closed with dental wax (Pluradent AG & Co KG).

Chickens from both groups were held in identical environmental and alimentary conditions (ambient temperature of 25°C with relative air humidity of 30%) during three weeks after hatching. An infrared lamp was an additional source of heat (35°C) for the chicks until day 14 post hatching. Food (complete feed, ssniff Spezialdiäten, Soest, Germany) and water were provided *ad libitum* to all animals.

### Body weight, fat and blood glucose

Post hatching, body weight was measured during three weeks until the day of experimental approach. At day of sacrifice (21.3 ± 0.2, n = 100), the birds were decapitated and blood and tissue samples were collected. Blood glucose was measured photometrically using the glucoseoxidase-peroxidase method (Dr Lange GmbH, Berlin, Germany). Body fat content was evaluated by drying the carcass mass (minus the stomach and intestine) to constant weight, followed by whole-body chloroform extraction in a Soxhlet apparatus [44]. Body fat was calculated as percentage of carcass mass.

### Sample preparation

For molecular biology analyses, the Nucleus infundibuli hypothalami (NI) was micro-dissected from deep frozen brain slices [45]. Genomic DNA and total RNA were simultaneously isolated from the NI brain probe using the ZR-Duet DNA/RNA MiniPrep Kit (Zymo Research, Irvine, CA, USA) according to the manufacturer’s instructions. cDNA was synthesized from total RNA according to the manufacturer’s protocol of the iScript cDNA Synthesis Kit (BioRad, Hercules, CA, USA), reverse transcriptase minus (RT-) negative controls were included. Genomic DNA was bisulfite treated using the EZ DNA Methylation-Gold Kit (Zymo Research) following manufacturer’s protocol.

### Gene expression analysis

Quantitative real-time PCR (qPCR) was used to measure the relative mRNA expression for the genes: insulin receptor (*INSR*), leptin receptor (*LEPR*), and glucose transporter 1 and 3 (*GLUT1*, *GLUT3*). Commercially available TaqMan probe-based gene expression assays were used (Life Technologies) and were run on an Applied Biosystems 7500 instrument according to the manufacturer’s protocol. Expression levels were normalized to the housekeeping gene *BETA ACTIN*. When possible exon-spanning primer sets were selected and qPCR was performed as duplex qPCR with housekeeping gene. Expression assays were performed in triplicate and relative gene expression of target genes was calculated using the 2^-ΔCT^ method corrected for the amplification efficiency calculated from standard curves of each primer set [45, 46]. TaqMan gene expression assays: *INSR*: Gg03330786_m1, *LEPR*: Gg03347016_m1, *GLUT1*: Gg03367103_m1, *GLUT3*: Gg03349364_m1 (all FAM-labeled), and *BETA ACTIN*: Gg03815934_s1, VIC-labeled, primer limited.

### DNA methylation assays

Amplicon regions for pyrosequencing analyses were chosen using UCSC genome browser (build: Chicken Nov. 2011, ICGSC Gallus_gallus-4.0/galGal4) based on the proximity of a CpG island to the transcriptional start site, the number of CpG sites and obtaining optimal PCR amplification and sequencing conditions. CpG islands annotated in UCSC browser were further confirmed with CpGPlot (http://www.ebi.ac.uk/Tools/seqstats/emboss_cpgplot/). Methylation assays were designed using the PyroMark Assay Design Software 2.0 (Qiagen, Valencia, CA, USA, www.qiagen.com). Bisulfite-converted DNA was mixed with 0.2 μM of each primer and amplified using HotStarTaq plus Master Mix (Qiagen) or ZymoTaq (Zymo Research) following standard procedures. Pyrosequencing was run on amplified PCR products using the Pyromark Q24 pyrosequencer (Qiagen). Percent methylation was individually analyzed across all individual CpG sites located within the following promoters/CpG island regions of interest: *INSR* (14 CpG sites), *LEPR* (7 CpG sites), *GLUT1* promoter (11 CpG sites), and *GLUT3* promoter (6 CpG sites). Each assay included a bisulfite conversion check to verify full conversion of the DNA and assays were validated with a methylation scale (0–100%). Primer sequences and pyrosequencing assay information are given in Table 1.

**Table 1:** Pyrosequencing assay information.

Target region	Primers	5’-3’ sequence	Chromosomal location*	Tm [°C]
***INSR***	Forward	ATTTATTTGTTGGAATTTATGATGTATTT	chr28:3,897,767–3,898,068	56.4
	Reverse Biotinylated	ACACTCAACTCTATCCCTTCTC		60.1
	Sequencing #1	GGATATATTAGTTGTTGTGGATG		
	Sequence to analyse #1	AYGTGGTAGTTYGATTYGYGGGYGTTTTTGTATTTGTTGTGTAGTTTTT		
	Unconverted sequence #1	ACGTGGCAGCCCGACTCGCGGGCG		
	Sequencing #2	TGGTAGAAGTTGAAGG		
	Sequence to analyse #2	TGAYGTAGYGTTAGTTTTYGAAGYGGTAGTGTTYGGGTGGGTAYGT		
	Unconverted sequence #2	TGACGCAGCGCCAGCCCTCGAAGCGGTAGTGCCCGGGTGGGCACGT		
	Sequencing #3	GAGGTATTTATTGTGATAGTAT		
	Sequence to analyse #3	TGTTYGTTYGAYGTGTAGTTTTGGGATTTGTAGG		
	Unconverted sequence #3	CTGCCCGTCCGACGTG		
***LEPR***	Forward	TTTAGGTAGGGGGTGGGGTTAAG	chr8:27,220,956–27,221,293	61.8
	Reverse Biotinylated	ATACTCCAAAACCCAACCCAACATAAAA		58.4
	Sequencing	TGAGAAGAGGAGTTGT		
	Sequence to analyse	TGTYGTTYGGTTYGTYGGAAATATGGYGGGTATTAAAGGTATYGAGYGTTGTYGGYGTTTYGT		
	Unconverted sequence	CCGCCCGGCCCGCCGGAAACATGGCGGGTATCAAAGGTACCGAGCGCTGCCGGCGCTCCGC		
***GLUT1***	Forward	AAGTTGTTTAGTAGGATGTAT	chr21:6,459,006–6,459,216	53.3
	Reverse Biotinylated	AACTTTCACCTCCCTAAAAACT		58.9
	Sequencing #1	GGAGGTGTGTTTGTT		
	Sequence to analyse #1	TTYGTAGTYGGTATYGTTYGGTTTTTATTTTTTG		
	Unconverted sequence #1	TCGCAGCCGGCACCGCTCG		
	Sequencing #2	GTTGTTTAGTAGGATGTATTAT		
	Sequence to analyse #2	TTYGGTYGGTTTAGGGYGGATGYGYGAGGYGGGAYGGAGGTGTGTTTGTTTT		
	Unconverted sequence #2	CGGCCGGCTCAGGGCGGATGCGCGAGGCGGGAC		
***GLUT3***	Forward	GAGTAGTATTAGTTATGGGTTGTATATTT	chr1:75,323,569–75,323,805	55.2
	Reverse Biotinylated	CCTTACCTTCTTATCAACCATCT		58.4
	Sequencing	GTTATGGGTTGTATATTTTTAA		
	Sequence to analyse	TTATTTTGGYGTTGGGGGAATYGAGGAGTTGGATYGTTYGTTATYGTTTYGGTTATTTTTTTTAYGGTTTTTTTTTT		
	Unconverted sequence	GCGTTGGGGGAACCGAGGAGCTGGACCGCCCGCCACCGCCCCGGCCACTCCTCCCACGGCTCCTCCTCTGTGCCCAACCCA		

* Chromosomal location is based on the UCSC Chicken Nov. 2011 (ICGSC Gallus_gallus-4.0/galGal4) Build.

Abbreviations: *GLUT*, glucose transporter; *INSR*, insulin receptor, *LEPR*, leptin receptor

### Statistical analyses

Investigations were part of a larger approach on metabolic, hormonal, morphometric, neuroelectrophysiological, and neurogenetic outcomes in prenatally glucose-treated chickens. For each parameter, obtained from randomly selected animals, the highest available number of sample measurements is presented here.

The baseline parameters (body weight, fat content and blood glucose) were calculated as means ± SEM. Real-time data are given as arbitrary units. For statistical analyses of the investigated peripheral parameters, real-time expression and pyrosequencing methylation data concerning differences between experimental groups Student´s t-test for independent samples (if normally distributed) or Mann-Whitney U-test (if not normally distributed) were used. Normal distribution of the data was tested before all statistical procedures using the Kolmogoroff-Smirnoff-Test. Differences in sex ratio were tested with chi-square test. Methylation and expression data were stratified by sex to determine any sex-specific effects. For analyses of relations between two variables, Spearman’s rank correlation test was performed overall and by groups.

Significance level was set at *p<*0.05. All statistical tests were carried out with GraphPad Prism (Version 4.03, San Diego, California, USA) and IBM SPSS for windows (Version 19.0, Munich, Germany), respectively.

## Results

### Body weight, fat and blood glucose

Body weight was measured at different time points of postnatal development and no significant differences were seen between groups during the entire observational period (Table 2). Accordingly, no difference was observed in total body fat content at day 21 between the NaCl-treated control group and the prenatally glucose-treated group (Table 2). Also, on day 21 of life, blood glucose levels did not differ between both groups (Table 2).

**Table 2:** Baseline characteristics, hypothalamic mRNA gene expression and overall promoter methylation in three-week-old chickens.

Variables	Prenatally NaCl-treated group	Prenatally glucose-treated group	*p*-value
**Sex ratio (M/F in %)**	28/72	36/64	0.532*
**Age at sacrifice (days)**	21.1 ± 0.2 (39)	21.4 ± 0.2 (61)	0.285
**Body weight development (g)**			
day 1	41.3 ± 0.7 (39)	42.7 ± 0.6 (61)	0.133
day 7	69.3 ± 1.1 (39)	67.7 ± 1.3 (61)	0.385
day 14	142.2 ± 2.5 (39)	137.6 ± 2.4 (61)	0.201
day of sacrifice	239.7 ± 4.1 (39)	238.3 ± 5.1 (61)	0.865
**Body fat content at day of sacrifice (%)**	8.2 ± 0.2 (15)	7.8 ± 0.2 (23)	0.200
**Blood glucose at day of sacrifice (mmol/l)**	9.3 ± 0.3 (15)	8.8 ± 0.2 (32)	0.253
**Hypothalamic mRNA gene expression (arbitrary units)**			
*INSR*	36.6 ± 3.1 (20)	25.6 ± 2.0 (25)	**0.006**
*LEPR*	0.45 ± 0.04 (20)	0.33 ± 0.02 (25)	**0.046**
*GLUT1*	427.6 ± 75.0 (20)	224.3 ± 28.6 (25)	**0.006**
*GLUT3*	337.5 ± 46.7 (20)	229.1 ± 29.4 (25)	**0.047**
**Hypothalamic promoter region methylation (%)**			
*INSR*	71.4 ± 0.9 (22)	70.9 ± 1.0 (26)	0.673
*LEPR*	0.7 ± 0.1 (19)	0.6 ± 0.1 (20)	0.432
*GLUT1*	11.9 ± 0.3 (22)	10.4 ± 0.6 (26)	0.100
*GLUT3*	1.4 ± 0.1 (20)	1.6 ± 0.1 (25)	0.187

Values are expressed as means ± SEM. Number of animals in parenthesis.

*p-*values were calculated using Student’s t-test or Mann-Whitney U-test when appropriate.

*chi-square test

Abbreviations: *GLUT*, glucose transporter; *INSR*, insulin receptor, *LEPR*, leptin receptor; M/F, males/females

### Gene expression analysis

**Fig 1:**
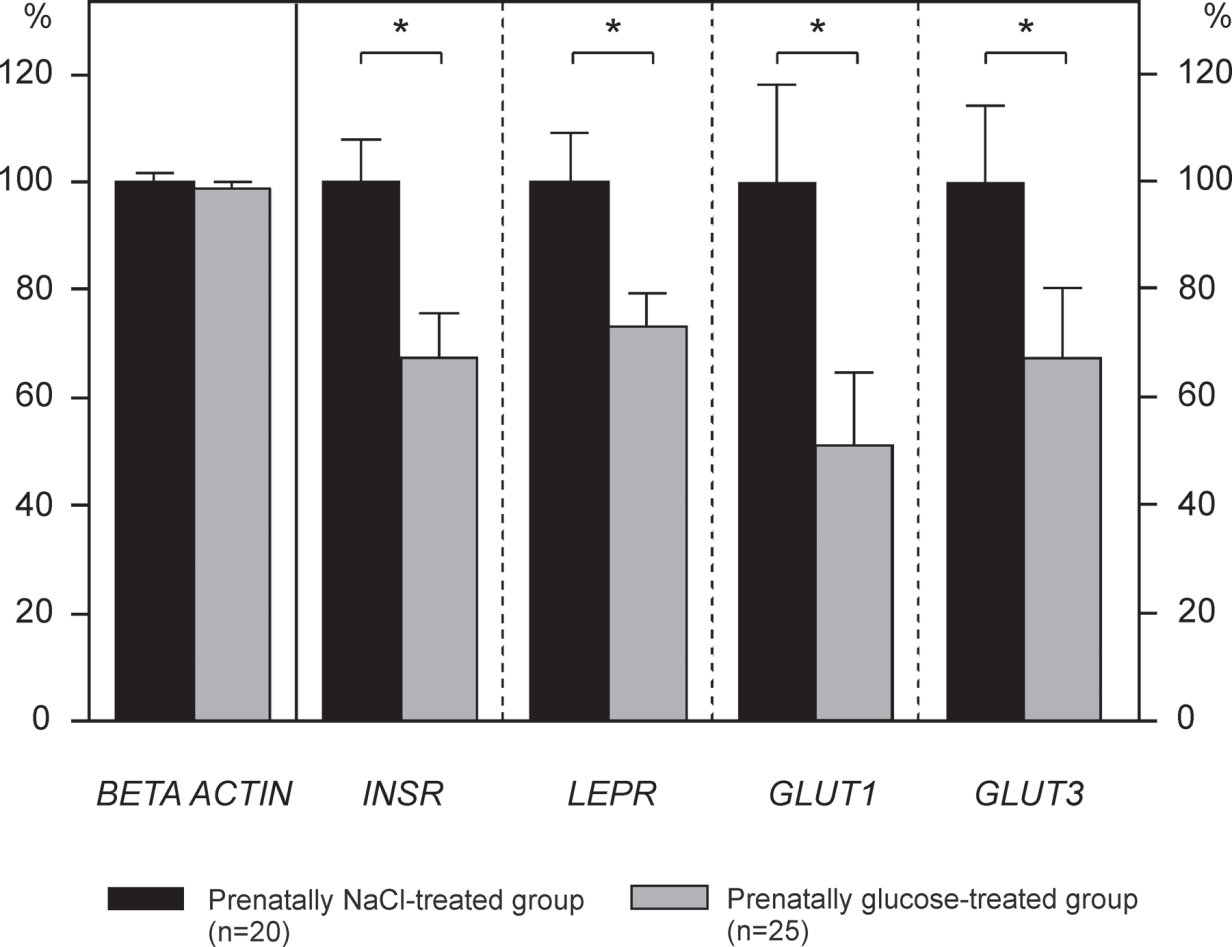
mRNA expression in the *Nucleus infundibuli hypothalami* (NI) in three-week-old chickens. Relative gene expression of insulin receptor (*INSR*), leptin receptor *(LEPR*), and glucose transporters 1 and 3 (*GLUT1*, *GLUT3*), all normalized to *BETA ACTIN*. Data are given as means ± SEM, shown as percentage of prenatally NaCl-treated controls. * *p*<0.05 (Mann-Whitney U-test)

As an experimental precondition [47], we first verified that mRNA expression of the housekeeping gene *BETA ACTIN* was unaffected by respective prenatal treatment. Prenatal glucose treatment did not lead to changes of *BETA ACTIN* mRNA expression in the *Nucleus infundibuli hypothalami* (NI) (Fig. 1).

However, three-week-old chickens prenatally treated with glucose showed a significant reduction of *INSR* and *LEPR* mRNA by approximately one-third as compared with prenatally NaCl-treated controls (all *p*<0.05, Table 2, Fig. 1). Additionally, both expression levels of *GLUT1* mRNA as well as *GLUT3* mRNA were significantly reduced in the prenatally glucose-exposed group (Table 2, Fig. 1).

### DNA methylation assays

**Fig 2:**
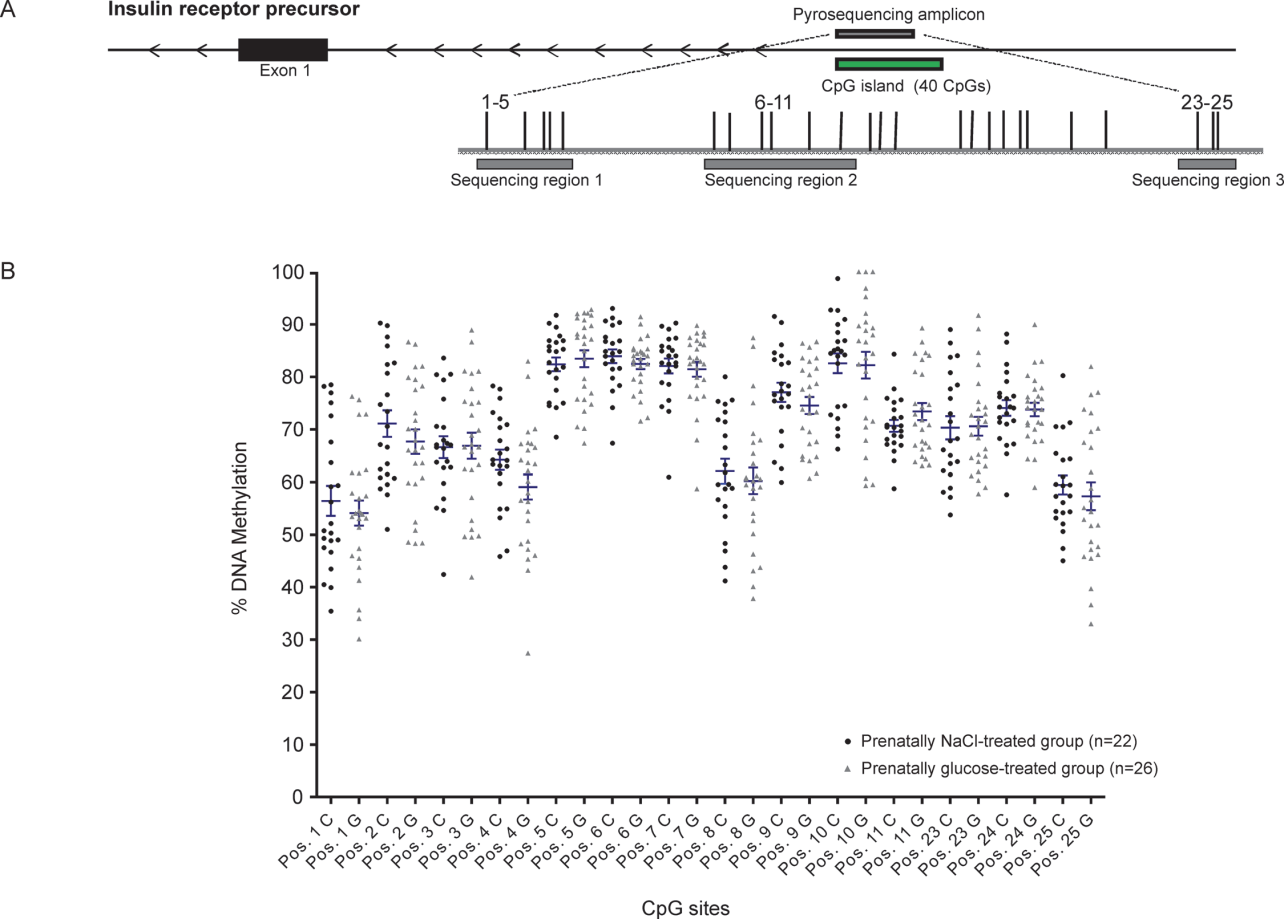
Insulin receptor (*INSR*). Schematic illustration of sequence map of the insulin receptor (*INSR*) gene promoter region with chromosomal location of pyroassay **(A)**, and corresponding DNA methylation levels at individual CpG sites for prenatally NaCl-treated controls (black) and the prenatally glucose-treated group (gray) **(B)**.

**Fig 3:**
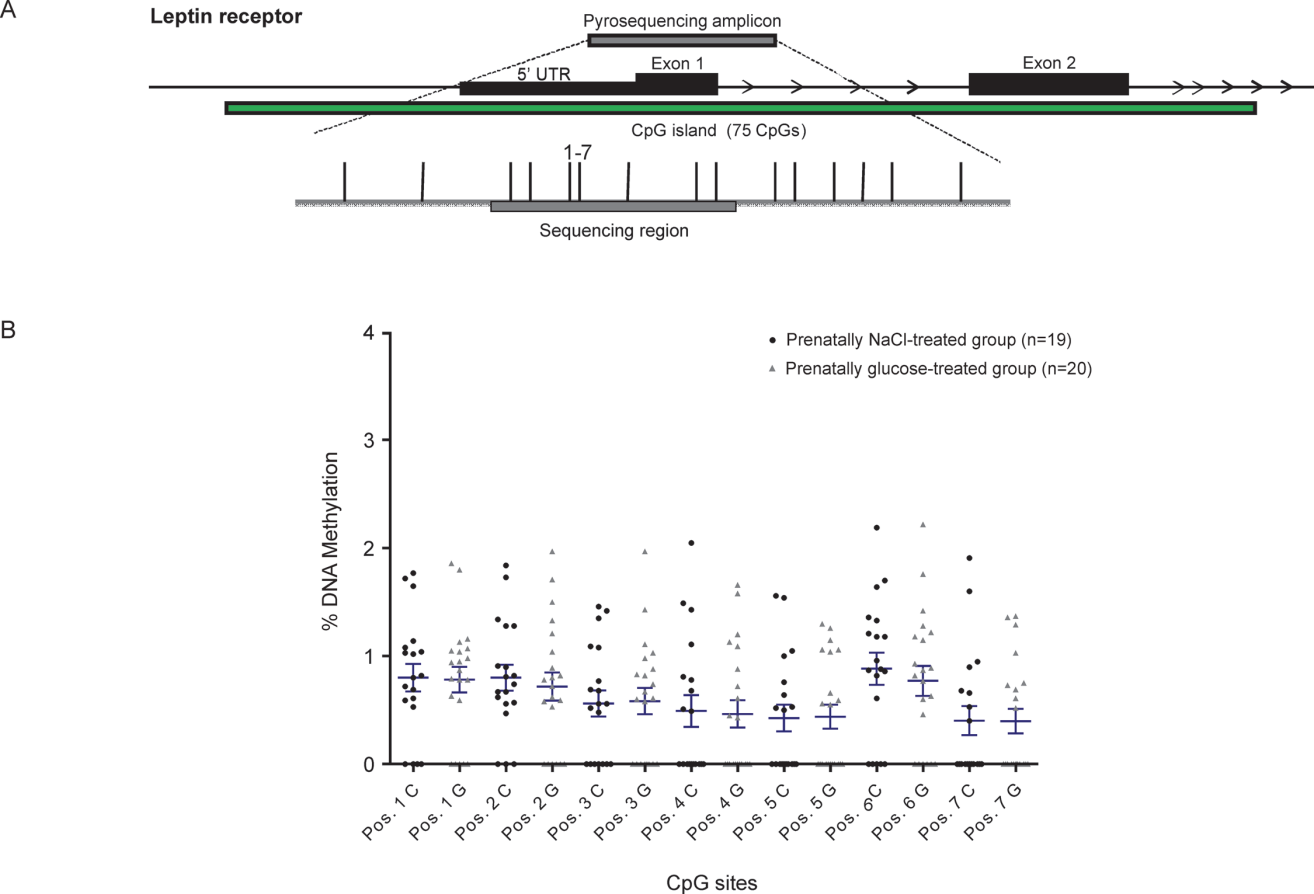
Leptin receptor (*LEPR*). Schematic illustration of sequence map of the leptin receptor (*LEPR*) gene promoter region with chromosomal location of pyroassay **(A)**, and corresponding DNA methylation levels at individual CpG sites for prenatally NaCl-treated controls (black) and the prenatally glucose-treated group (gray) **(B)**.

**Fig 4:**
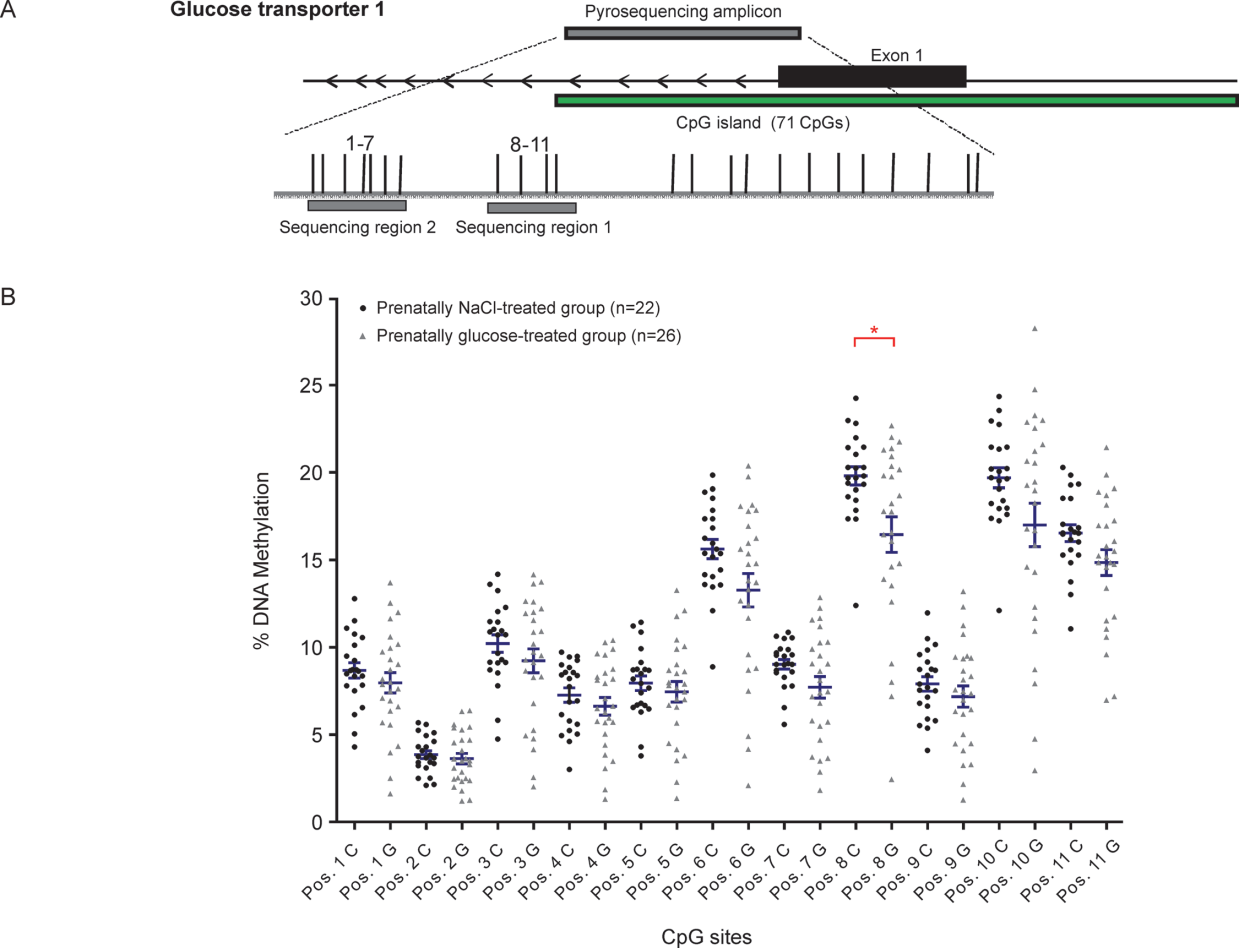
Glucose transporter 1 (*GLUT1*). Schematic illustration of sequence map of the glucose transporter 1 (*GLUT1*) gene promoter region with chromosomal location of pyroassay **(A)**, and corresponding DNA methylation levels at individual CpG sites for prenatally NaCl-treated controls (black) and the prenatally glucose-treated group (gray) **(B)**.* *p*<0.05 (Mann-Whitney U-test)

**Fig 5:**
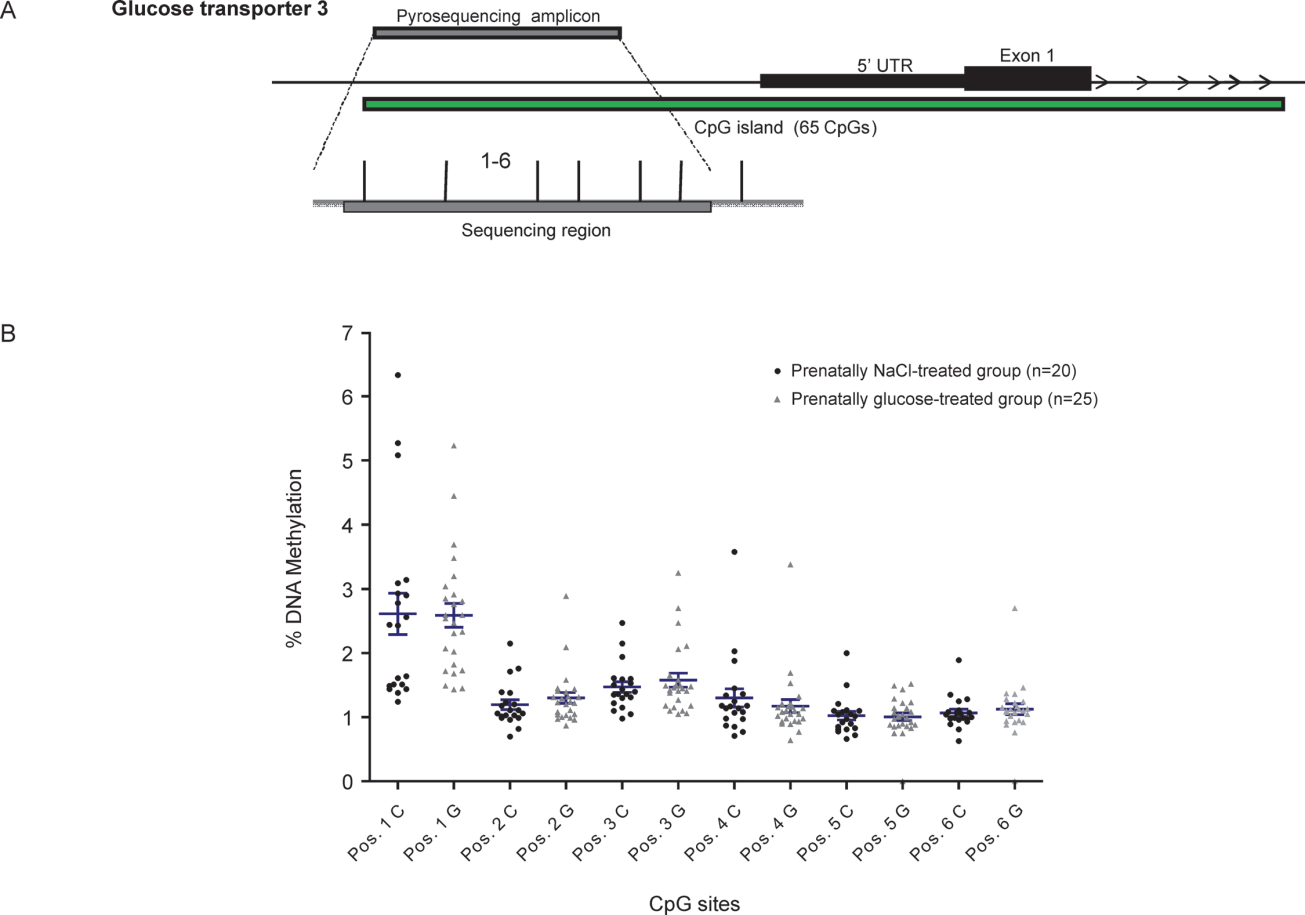
Glucose transporter 3 (*GLUT3*). Schematic illustration of sequence map of the glucose transporter 3 (*GLUT3*) gene promoter region with chromosomal location of pyroassay **(A)**, and corresponding DNA methylation levels at individual CpG sites for prenatally NaCl-treated controls (black) and the prenatally glucose-treated group (gray) **(B)**.

Investigations at the promoter regions of the genes of interest showed no disruption of methylation levels occurred due to prenatal high-glucose exposure, neither in total percentage of promoter DNA methylation (Table 2) nor at single CpG sites (Figs. 2–5). A weak but statistically significant difference according to group was found only at one out of a total of 38 CpG sites investigated per animal, (CpG site 8 in *GLUT1*: NaCl: 19.8% ± 0.5 *vs*. Glc: 16.5% ± 1.0, *p*<0.05, Fig. 4).

### Correlation analyses and sex specific observations

**Fig 6:**
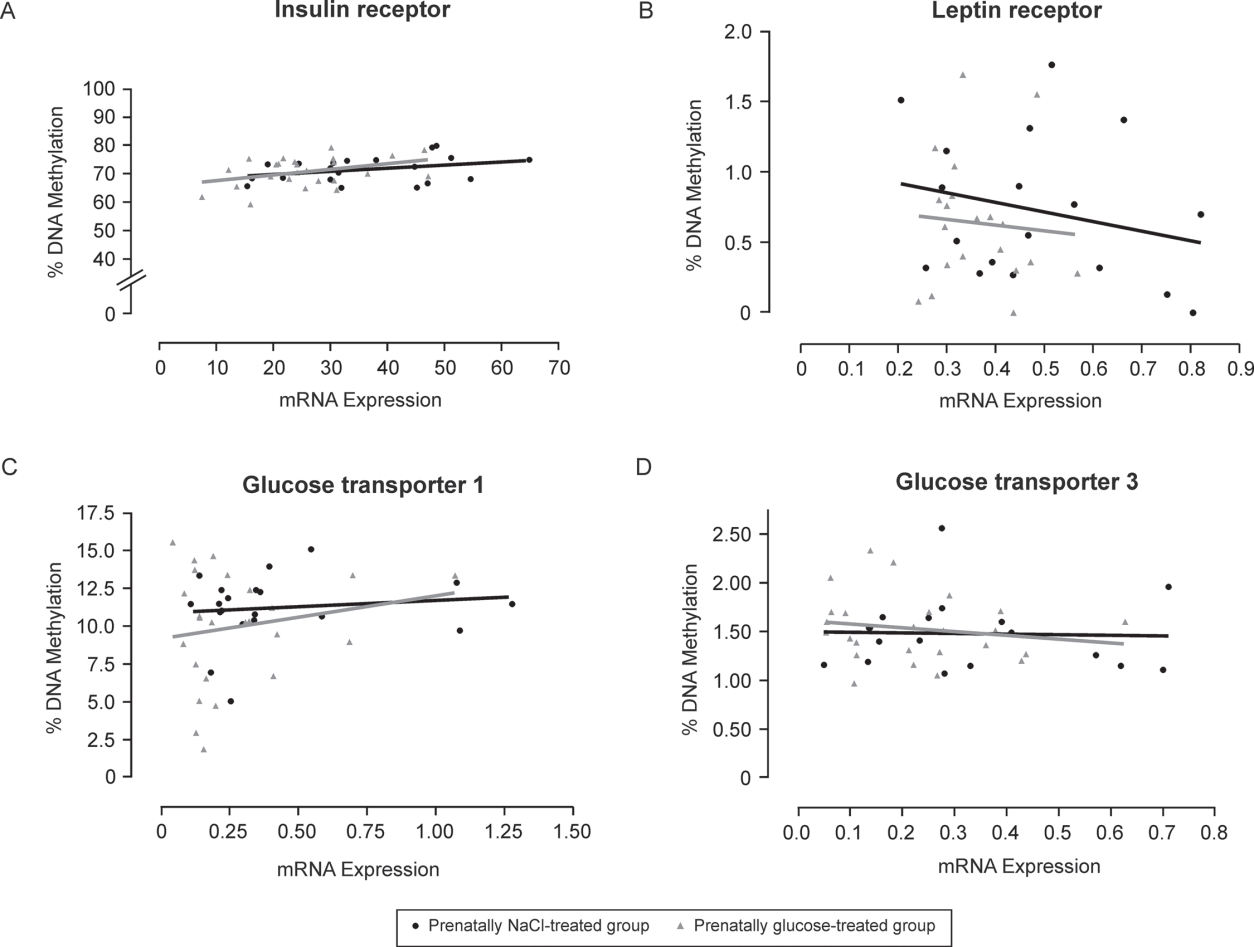
Correlation analyses. Relations between total DNA methylation levels and corresponding mRNA expression of the insulin receptor **(A)**, leptin receptor **(B)**, glucose transporter 1 **(C)** and glucose transporter 3 **(D)** in the *Nucleus infundibuli hypothalami* (NI) in three-week-old chickens of prenatally NaCl-treated controls (black) as compared to the prenatally glucose-treated group (gray).

The expression of glucose transporter genes, *GLUT1* and *GLUT3*, showed positive overall correlation (r = 0.654, *p*<0.0001). Interestingly, *GLUT1* and *GLUT3* expression also showed positive overall correlation with *LEPR* expression (r = 0.530, *p* = 0.0002; r = 0.553, *p*<0.0001, respectively). Similarly, *GLUT1* and *INSR* expression were significantly positively correlated across the cohort (r = 0.386, *p*<0.01) and a similar trend was also seen for *GLUT3* and *INSR* (r = 0.170, *p* = 0.265). However, none of the candidates under investigation had mRNA expression levels significantly correlating with levels of promoter methylation, neither to overall methylation average across a pyrosequencing region (Fig. 6) or at individual CpG sites. Spearman coefficients by group for methylation *vs*. mRNA were as follows: *INSR* (NaCl: r = 0.388, Glc: r = 0.251), *LEPR* (NaCl: r = -0.090, Glc: r = -0.186), *GLUT1* (NaCl: r = -0.022, Glc: r = 0.101), and *GLUT3* (NaCl: r = -0.159, Glc: r = -0.076; all *p*-values are not significant; Fig. 6). Even methylation levels at the only altered CpG site, CpG 8 in the *GLUT1* promoter region, and *GLUT1* mRNA expression showed no correlation (r = 0.07, *p* = 0.633).

Although sex ratio did not differ between groups (Table 2), we finally addressed potential sex differences by group at the molecular level, too. Analyses of *GLUT3* and *LEPR* mRNA expression data stratified by sex revealed no sex-specific pattern. The only statistically significant differences in gene expression between groups and by sex were observed in males for *GLUT1* (NaCl: 495.6 ± 118.4 *vs*. Glc: 204.4 ± 39.2) and *INSR* (NaCl: 40.9 ± 4.8 *vs*. Glc: 25.6 ± 3.3) (all *p*<0.05). However, overall group differences for *GLUT1* and *INSR* remained when adjusted for sex. The one and only difference in methylation levels, occurring at CpG site 8 for *GLUT1*, was rather linked to females (NaCl: 20.1% ± 0.5 vs. Glc: 15.3% ± 1.6, *p*<0.05) however, the overall group difference was no longer significant when adjusted for sex.

## Discussion

There are a variety of animal models on ‘perinatal programming’, examining long-term effects of altered intrauterine and early postnatal conditions for later health risks, especially concerning diabetes and obesity. Identifying concrete causal factors, however, is rather difficult due to the complexity of the interaction between the fetus, the maternal organism, and environmental factors. Therefore, we aimed to investigate consequences of short-term exposure to high glucose within the critical period of late prenatal development on respective postnatal hypothalamic gene expression in the avian embryo model representing a unique ‘closed system’ to examine and identify risk factors under well-controlled and highly standardized conditions. The study demonstrates that a strong direct relationship exists between prenatal high-glucose exposure and altered subsequent hypothalamic gene expression.

In the human, it is well known that materno-fetal hyperglycemia, such as occurring in gestational diabetes (GDM), can have long lasting deleterious effects on offspring’s health. Epidemiological studies have shown that offspring of mothers with diabetes during pregnancy have an increased risk for developing diabetes, obesity and associated metabolic and cardiovascular diseases later in life, even irrespective of genetic predisposition [48–50]. However, whether increased glucose itself or rather accompanying alterations of lipids, amino acids and/or hormone concentrations are responsible for acquired malprogramming of body weight regulation and metabolism remain unclear. Interestingly, epidemiological studies strongly indicate a particular dose-dependent impact of glucose/hyperglycemia for the overall outcome of offspring of diabetic mothers [51]. On the other hand, offspring of obese pregnant women without having gestational diabetes are also at increased risk for an adverse outcome suggesting that components other than glucose may also play a crucial role [52]. Both GDM and obesity appear to be independently associated with adverse pregnancy outcomes, but their combination has a greater impact than one alone [52].

Numerous experimental observations have shown that materno-fetal hyperglycemia in mammals may lead to later increased disposition to hyperphagia, hyperinsulinemia, impaired glucose tolerance and overweight in exposed offspring [36, 53, 54]. Structural and functional malorganization of hypothalamic regulatory circuits has been identified as a key mechanism [36, 37, 55]. Interestingly, these hypothalamic alterations were preventable by normalization of gestational hyperglycemia [54]. Similarly, both neonatal exposure to maternal diabetes as well as neonatal overfeeding causes a ‘malprogramming’ of hypothalamic insulinergic and leptinergic pathways in the offspring [40, 56–58]. The acquired phenotype in these models has been linked to reduced responsiveness to the satiety signals leptin and insulin in arcuate hypothalamic neurons of juvenile as well as adult offspring [34, 35].

Here, we could demonstrate reduced receptor expression (*INSR*, *LEPR*) in three-week-old chickens, exposed only to elevated glucose prenatally. Interestingly, while little is known on leptinergic action in the chicken hypothalamus insulinergic function has clearly been demonstrated [14]. As in mammals, decreased action of the satiety-signals, insulin and leptin, at the mediobasal hypothalamus appears to favor activation of orexigenic/catabolic pathways and respective neuropeptide expression [10, 14]. Moreover, the *INSR* and *LEPR* findings were accompanied here by clear reduction of *GLUT1* and *GLUT3* expression. *GLUT1* is the main glucose transporter at the blood-brain-barrier in mammals [59] while, the majority of glucose sensing neurons use glucose transporter 3 (*GLUT3*) as primary transporter. These functions of brain *GLUT1* and *GLUT3* seem to be very similar in birds as in mammals [31–33]. Our data, therefore, appears to indicate an acquired ‘diabesogenic’ predisposition at the hypothalamic mRNA expression level of prenatally high-glucose exposed chicken.

Prenatally glucose-exposed chicken in the present study showed a significant decrease of *INSR*, *LEPR*, *GLUT1* and *GLUT3*, while not showing differences in plasma glucose levels, body weight and total body fat as compared to the prenatally NaCl-exposed controls. Therefore, suppression of hypothalamic anorexigenic pathways appears not to be caused here by a negative feedback regulation within the current metabolic state but might rather be a long-term consequence of temporary prenatal hyperglycemia. The lack of phenotypic ‘obesogenic’ alterations, however, might indicate that early ‘programming’ of ‘obesity’ depends on additional exposures rather than prenatal hyperglycemia itself. General materno-fetal overfeeding, in addition to elevated glucose itself, might be crucial here, translationally in line with the ‘mixed nutrients hypothesis’ [60] and observations in offspring of overweight/obese women irrespective of GDM [52]. At the same time, this may illustrate the mechanistic value of investigations in the chicken model for the ‘perinatal programming of diabesity’ field. Note, while our focus was primarily on the programming linked to the critical brain region associated with physiological/metabolic pathways and at an earlier time point of life (3 weeks of age), we understand that phenotypic alterations could become more prominent only in later age. For instance, investigations in genetic crosses of high and low weight chicken lines, without introducing any treatment, have reported differences in baseline parameters only at later time points (>5–11 weeks) and in specific peripheral tissues (esp. abdominal fat) without seeing changes in overall body weight [10, 61]. Normal total body weight and even total body fat in chicken does not necessarily exclude a ‘diabesogenic’ phenotype especially since abdominal fat depots seem to be particularly affected in this species [10, 61]. Finally, in rats and humans, phenotypic expression of perinatally acquired diabesity disposition has translationally be shown to occur only at later juvenile and adult age or even after an early age ‘recovery’ [36, 62, 63].

Surprisingly, clearly altered expression of *INSR*, *LEPR*, *GLUT1* and *GLUT3* did not coincide with nor were explainable by alterations of respective promoter DNA methylation patterns as indicated with a lack of negative correlation between gene expression and methylation levels at either individual CpG sites or overall average across a promoter region amplicon (Fig. 6). Accordingly, further reflection on potential causes arise from the methodological design of our study and/or mechanisms other than altered promoter methylation acquired through prenatal high-glucose exposure. Regarding the genomic regions selected for methylation analyses, most of the investigated promoter region CpG islands encompassed the 5’UTR, as well as the first exon and intron (*LEPROT*, *GLUT1* and *GLUT3*), with the exception of the CpG island located approximately 2 KB upstream of the start of *INSR*. The chicken *INSR* locus, in particular, is not well annotated and under-examined in comparison to mammals. Conservation across species (*e*.*g*. human and rodent) was therefore considered and revealed a substantial lack of conservation at these chicken CpG island regions. It was previously reported that chicken *GLUT1* and *GLUT3* have 80.4% and 70.4% amino acid sequence homology with human *GLUT1* and *GLUT3*, respectively [64]. Chicken and human *LEPR* have 47.2% amino acid sequence homology [29] and the *INSR* amino acid sequence homology of chicken and human depends on the domain investigated [65], with considerable high ranges of 69% up to 93% based on C-terminal, juxtamembrane and tyrosine kinase domains. Despite the degree of homology at the amino acid level, low to no conservation between human and chicken at 5’UTRs and start sites was observed and reported as compared to exon sequences [17]. Functional elements in the chicken-human alignment are reduced in contrast to the human-mouse-rat alignments [17]. An interesting suggested difference in the bird is the absence of the epigenetic phenomenon of genomic imprinting, with known imprinted genes in mammals showing biallelic expression in chicken [66, 67]. In addition, *DNMT3L*, which is essential for the establishment of imprinting marks as demonstrated in the mouse, has not been identified in chicken further implying that genomic imprinting as seen in mammals may not occur in birds [68, 69]. In general, Head *et al*. reported approximately 56% of CpG sites in the chicken brain are methylated, versus approximately 70% in mammals [70]. On the other hand, while there is a growing interest in the epigenetic patterns in the chicken [71], not much has been reported so far on methylation levels at gene specific promoter regions examined in our study. Recently, Nätt *et al*. addressed potential brain promoter methylation related sex differences according to behavior, and observed few [72]. Authors reported even up-regulation of the Zinc Finger RNA binding protein (*ZHR*) gene despite hypermethylation, which they discussed, might indicate a novel epigenetic regulatory mechanism CG independent or, perhaps, could be from non-CpG-sequence methylation. Non-CpG methylation was observed to be particularly prevalent in the adult human and in mouse brain [73] and specifically more abundant in neuronal compared with non-neuronal cells [74] however; non-CpG methylation has yet to be fully investigated in the bird. A growing consideration is that non-CpG methylation could provide insight in understanding the mechanisms of cell-type-specific gene expression in the brain.

Beyond methylation-related gene expression and gene expressivity, chromatin structure and looping of locus control regions (involving enhancer elements) regulate chicken genes with distinct temporal expression patterns. Additionally, histone modifications in respective domains (*e*.*g*. *BETA-GLOBIN*) are thought to modulate the ‘open’ or ‘closed’ chromatin conformation allowing for enhancer elements to act to regulate expression in a tissue/cell-type and developmental time point specific manner [75]. These mechanisms appear to be relevant translationally, *i*.*e*., over species, but not comprehensively characterized so far in birds. Non-coding RNA and anti-sense transcripts could be acting to regulate expression. Non-coding RNAs are conserved in the bird which could hint to their mechanistic role having greater importance in chicken compared to other species [17].

Finally, beyond addressing above mentioned alternative epigenomic aspects in future studies, potential microstructural as well as regulatory causes should also be considered when interpreting our observations. First, as indicated by respective positive correlations *GLUT1* and *GLUT3* mRNA decrease might be a result, at least in part, of decreased *INSR* and *LEPR* expression. *GLUT* expression regulation downstream of the insulin- and leptin receptor is well established [31–33, 59], while acquired hypothalamic resistance to insulin and leptin, resulting from pre- and perinatal high-glucose exposure and overfeeding, has been shown in rats [34–37] and functionally been indicated even in the human [38]. Also, microstructural disorganization has been proposed and shown to be a second critical and general mechanism of prenatal, especially central nervous, imprinting and programming [4, 76]. For instance, final number and numerical density of neurons in mediobasal hypothalamic nuclei have been shown to become altered through prenatal exposure to elevated glucose levels [36, 37, 57, 62, 77], potentially contributing to overall altered mRNA levels in the ARC/NI. Though the primary focus of this study was the ARC/NI, we do suggest that neighbouring regions, e.g. ventromedial hypothalamic nucleus, [37, 77–82] could also be affected by prenatal high-glucose exposure, and maybe even with epigenomic modifications.

In summary, temporary exposure to high glucose levels in late prenatal life of chicken, irrespective of and without any other developmental alterations, gave rise to reduced *INSR*, *LEPR*, *GLUT1* and *GLUT3* mRNA expression in the ARC/NI hypothalamic region. This speaks for an acquired alteration of the molecular ‘set-point’ and continuous suppression through prenatal exposure to hyperglycemia. Data therefore may indicate in a translational sense that elevated glucose acts as a ‘metabolic disruptor’ during central nervous development, leading to a persistent malprogramming at the expression level of candidate genes addressed here. At the same time, absence of related changes in promoter DNA methylation seems to challenge an over-simplified favoring of respective promoter epigenomics as the principle and only mechanism in prenatal imprinting and central nervous programming.
